# The relevance, biases, and importance of digitising opportunistic non-standardised collections: A case study in Iberian harvestmen fauna with BOS Arthropod Collection datasets (Arachnida, Opiliones)

**DOI:** 10.3897/zookeys.404.6520

**Published:** 2014-04-24

**Authors:** Izaskun Merino-Sáinz, Antonio Torralba-Burrial, Araceli Anadón

**Affiliations:** 1 Departamento de Biología de Organismos y Sistemas, Universidad de Oviedo. C/ Catedrático Rodrigo Uría s/n 33071 Oviedo, Asturias, España; 2 Cluster de Energía, Medioambiente y Cambio Climático, Campus de Excelencia Internacional, Universidad de Oviedo, España

**Keywords:** Biodiversity collections, entomological collections, digitisation priorities, sampling methodology, biases, Opiliones, distribution, Iberian Peninsula

## Abstract

In this study, we analyse the relevance of harvestmen distribution data derived from opportunistic, unplanned, and non-standardised collection events in an area in the north of the Iberian Peninsula. Using specimens deposited in the BOS Arthropod Collection at the University of Oviedo, we compared these data with data from planned, standardised, and periodic collections with pitfall traps in several locations in the same area. The Arthropod Collection, begun in 1977, includes specimens derived from both sampling types, and its recent digitisation allows for this type of comparative analysis. Therefore, this is the first data-paper employing a hybrid approach, wherein subset metadata are described alongside a comparative analysis. The full dataset can be accessed through Spanish GBIF IPT at http://www.gbif.es:8080/ipt/archive.do?r=Bos-Opi, and the metadata of the unplanned collection events at http://www.gbif.es:8080/ipt/resource.do?r=bos-opi_unplanned_collection_events. We have mapped the data on the 18 harvestmen species included in the unplanned collections and provided records for some species in six provinces for the first time. We have also provided the locations of *Phalangium opilio* in eight provinces without published records. These results highlight the importance of digitising data from unplanned biodiversity collections, as well as those derived from planned collections, especially in scarcely studied groups and areas.

## General description

**Purpose:** Existing knowledge on the distribution of harvestmen throughout the Iberian Peninsula is still highly fragmented (Prieto 2003). Several studies on particular genera (e.g., Prieto 2004, Prieto and Fernández 2007, Merino-Sáinz et al. 2013a), as well as studies with planned, repeated, and systematic samplings in some locations (Merino-Sáinz and Anadón 2008, 2013, Rosa García et al. 2009a, b, 2010a, b, Merino-Sáinz et al. 2013b) have contributed to improving this knowledge. Global or specific studies on biodiversity are also enabled by the review, digitisation, and data release of specimens housed in biodiversity collections at research centers, universities, museums, and in the possession of individuals. These practices facilitate the identification of gaps in our knowledge of taxa distribution across space and time.

Within this context, biodiversity data on specimens from the BOS Arthropod Collection (hosted at the Department of Organisms and Systems Biology, (Spanish acronym BOS), University of Oviedo) are being digitised and the data released through the Global Biodiversity Information Facility (GBIF) data-portal (Department information as data published and available datasets: http://www.gbif.org/publisher/95cb537c-74c5-4c1e-ae24-32e7ea08f380; general digitisation and data release workflow of the BOS Arthropod Collection: Torralba-Burrial and Ocharan 2013). However, there is a need to establish priorities in the digitisation of specimens data of biodiversity collections (see Berents et al. 2010 for different approaches), especially in situations where mass digitisation methods are not available (see Beaman and Cellinese 2012). As such, we evaluate whether the effort of reviewing and digitising (harvestmen) specimens from unplanned collection events can provide useful data on their biodiversity and distribution, or whether it is better to limit digitisation to only those specimens associated with standardised samplings (planned collection events), which provide quantitative data in each location and allow for comparisons between locations over time.

The BOS Arthropod Collection includes harvestmen from the northern part of the Iberian Peninsula that, since 1977, have been obtained through systematic repeated sampling in several locations, as well as through non-harvestmen-specific sampling and accidental occurrences. Specimens were collected systematically from the Muniellos Biosphere Reserve between 2000 and 2002 (Merino-Sáinz and Anadón 2008), and from several locations in the provinces of Asturias, Cantabria, and Pontevedra between 2009 and 2011 (Merino-Sáinz et al. 2013c describe the harvestmen subcollection of BOS). Therefore, we decided to study and compare the data derived from unplanned collections events (untargeted sampling) with these data derived from planned, standardised, and periodic sampling. We have combined these analyses with the published results of similar studies using pitfall traps in western Asturias (Rosa García et al. 2009a, b, 2010a, b). In effect, this is the first data-paper to employ a hybrid approach, wherein the subset metadata from a large published dataset are described and a comparative analysis is carried out, in order to evaluate digitisation priorities. The aims of this paper are, thus, to 1) test whether the effort of reviewing and digitising (harvestmen) specimens from unplanned collection events can provide useful data about their distribution and biology, and 2) assess possible biases arising from the use of this type of data.

## Project details

**Project title:** Informatización de la Colección de Artrópodos BOS de la Universidad de Oviedo / Digitisation of the BOS Arthropod Collection of University of Oviedo

**Personnel digitisation and metadata creator:** A. Torralba-Burrial

**Administrative contact:** A. Anadón

**BOS-Opi determination specialist:** I. Merino-Sáinz

**Subset collectors:** Most of the collectors provided less than ten harvestmen records in this subset. Only Merino-Sáinz collected more than 10 specimens. All of the collectors are listed in Supplementary material 1 (http://hdl.handle.net/10651/24734) next to each specimen.

**Funding:** The digitisation of this biological collection was supported by the Spanish National R+D+i Plan (MICINN, Spanish Government, grant ref. PTA2010-4108-I) and PCTI Asturias (Asturias Regional Government, ref. COF11-38) through a contract with ATB.

Specimens were identified by IMS, which was supported by a Severo Ochoa pre-doctoral grant (ref. BP08039, FICYC, Asturias Regional Government).

**Figure 1.**
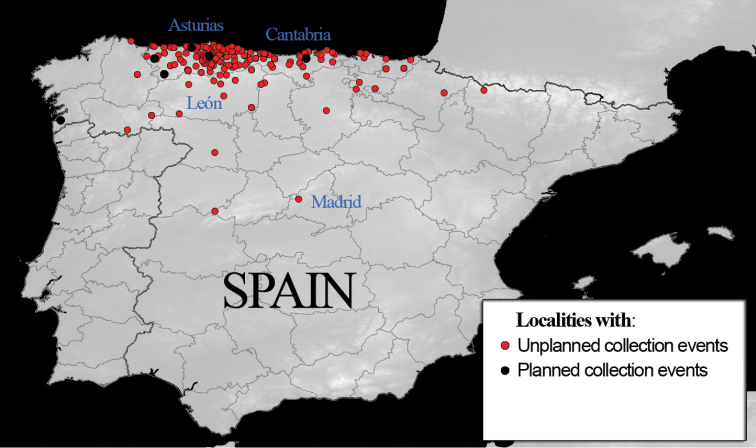
Distribution of specimens included in this subset.

**Study area descriptions/descriptor:** Harvestmen data in the subset are from the same area as the full Opiliones of the BOS Arthropod Collection dataset. Specimens are mainly from the northern third of the Iberian Peninsula (chiefly the Spanish provinces of Asturias, Cantabria, and León, with a few records from other neighbouring provinces) (see Figure 1).

Data sources of harvestmen data from planned collection events with pitfall trapping: Merino-Sáinz and Anadón (2008, 2013); Merino-Sáinz et al. 2013c, Rosa García et al. (2009a, b, 2010a, b).

**Figure 2.**
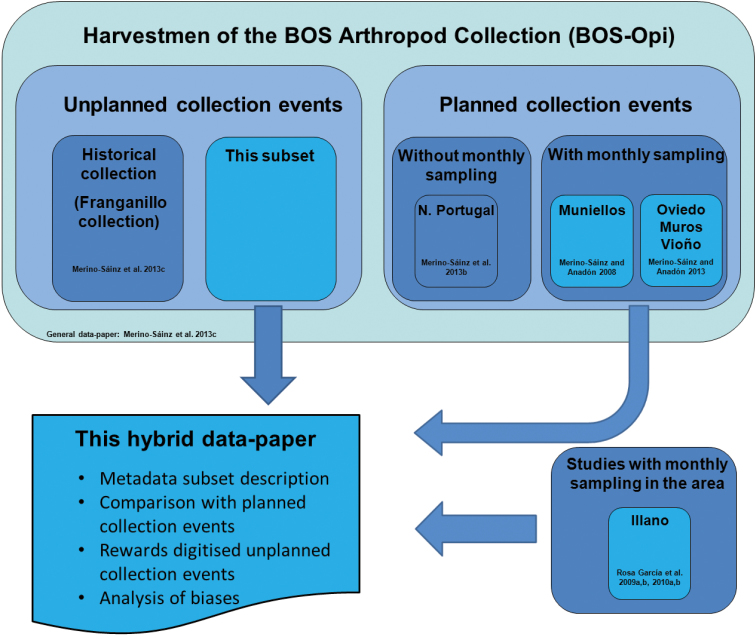
A diagram depicting the methodological design of this hybrid data paper. Harvestmen in the BOS Arthropod Collection (Merino-Sáinz et al. 2013c) have come from several sources: some from unplanned collection events and some from planned collections. For this hybrid data-paper, we compared the data subset of unplanned collection events with the subsets of harvestmen from planned collection events using monthly sampling (Merino-Sáinz and Anadón 2008, 2013), and the harvestmen of similar planned events in the same area (Rosa García et al. 2009a, b, 2010a, b). All of the subsets compared appear in light blue in the diagram.

**Design description:** The data subset is part of the large dataset of Opiliones housed in the BOS Arthropod Collection (Universidad de Oviedo 2013-). Details of the digitisation process are available in the description of the BOS Collection digitisation workflow (Torralba-Burrial and Ocharan 2013) and in the data paper on the harvestmen subcollection (Merino-Sáinz et al. 2013c). In that data-paper, we argue that the large dataset could be used to assess, among other things, the importance of unplanned collection data in filling in knowledge gaps if planned (standardised sampling) collection data are not available or are incomplete. With this aim in mind, we chose a data subset from the harvestmen subcollection, which included data only from unplanned collection events. This subset was used to compare specimens data with published data obtained through planned, standardised, and periodic samplings using pitfall traps in several locations in the north of the Iberian Peninsula (see Merino-Sáinz and Anadón 2013 for a checklist of the species found in the studies). Moreover, we used all of the published data on Iberian harvestmen, not just the BOS Arthropod Collection harvestmen data, to analyse the distributional knowledge gained by digitising this subset, e.g., the first provincial records. Figure 2 shows a diagram depicting the methodological design of our analysis.

## Taxonomic coverage

**General taxonomic coverage description:** Seventeen taxa were identified to the species level. Due to the biological phase or sex of the specimens, or unresolved taxonomic issues, 39 records (8%) were assigned only to the genus level. Those specimens belonging to the genus *Paramiopsalis* represent species number 18. The numbers of records per species and per family (also including specimens identified to the genus level) are shown in Table 1.

**Table 1.** Harvestmen families and species included in the data subset.

Family	Species	Abundance	Chorology
Sclerosomatidae	*Leiobunum blackwalli* Meade	129	EU
	*Leiobunum rotundum* (Latreille)	94	EU
	*Homalenotus laranderas* Grasshoff	28	EI
	*Gyas titanus* Simon	19	EU
	*Leiobunum* spp.	5	
	*Homalenotus quadridentatus* (Cuvier)	3	EU
	*Homalenotus* spp.	3	
**Total Sclerosomatidae**	**5**	**281**	
Phalangiidae	*Phalangium opilio* Linnaeus	109	HO
	*Odiellus* spp.	37	
	*Paroligolophus agrestis* (Meade)	16	HO
	*Dicranopalpus ramosus* (Simon)	13	EU
	*Odiellus simplicipes* (Simon)	10 ♂♂	EI
	*Odiellus seoanei* (Simon)	6 ♂♂	EI
	*Paroligolophus* spp.	5	
	*Odiellus spinosus* (Bosc)	2 ♂♂	EU
	*Megabunus diadema* (Fabricius)	4	EU
**Total Phalangiidae**	**7**	**202**	
Ischyropsalididae	*Ischyropsalis hispanica* Roewer	10	EI
Nemastomatidae	*Nemastomella dentipatellae* (Dresco)	8	EI
	*Nemastoma hankiewiczii* (Kulczynski)	1	EI
**Total Nemastomatidae**	**2**	**9**	
Trogulidae	*Trogulus* sp. aff. *nepaeformis* (Scopoli)	21	
	*Anelasmocephalus cambridgei* (Westwood)	1	EU
**Total Trogulidae**	**2**	**22**	
Sironidae	*Paramiopsalis* sp.	12	EI

EI: Iberian endemic, EU: European, HO: Holarctic (Merino-Sáinz and Anadón 2008)

The family Phalangidae comprised the largest number of identified species (seven), followed by Sclerosomatidae (five). However, when the number of records is considered, Sclerosomatidae was the most frequent family (around one hundred records for both *Leiobunum blackwalli* and *Leiobunum rotundum*), followed by Phalangidae, with only one species *Phalangium opilio* with a high number of records, similar to the *Leiobunum* species, and other species with only a few records. Five families (and the remaining species) had less than 30 records each.

## Taxonomic ranks

**Kingdom:** Animalia

**Phylum:** Arthropoda

**Class:** Arachnida

**Order:** Opiliones

**Family:** Sclerosomatidae, Phalangiidae, Ischyropsalididae, Nemastomatidae, Trogulidae, Sironidae

**Common names:** Animals, Arthropods, Arachnids, Harvestmen

## Spatial coverage

### General spatial coverage

Harvestmen specimens of this subset are mainly from the northern third of Spain, similar to spatial coverage of the large dataset (see Merino-Sáinz et al. 2013c for a wider overview).

### Coordinates

40°21'36"N and 43°40'12"N Latitude; 7°26'24"W and 0°31'12"W Longitude.

## Temporal coverage

1977–2012.

## Natural collections description

**Parent collection identifier:** Colección de Artrópodos BOS

**Collection name:** Colección de Artrópodos BOS de la Universidad de Oviedo: Opiliones (BOS-Opi) subset recolecciones no planeadas

**Collection identifier:** http://www.gbif.org/dataset/7cebf715-c3b0-4477-99e7-f6f3aca27bbe

**Curatorial unit:** 472 with an uncertainty of 0 (Data records)

**Curatorial unit:** 536 with an uncertainty of 0 (Specimens)

## Methods

**Method step description:** This data subset was extracted from the large dataset of harvestmen in the BOS Arthropod Collection (University of Oviedo 2013-). Specimens data in the subset are listed in Supplementary material 1 - Appendix A (http://hdl.handle.net/10651/24734), which includes the municipality, location, date, sampling method, amount, sex, and collector of the 536 taxonomically identified specimens corresponding to BOS-Opi codes 493-960. Using these codes, most of the specimen data (including their georeferenced locations) are available in reusable format in the DarwinCore Archive of the data-paper describing the BOS-Opi subcollection (Merino-Sáinz et al. 2013c) and through the GBIF data-portal (Universidad de Oviedo 2013-, http://data.gbif.org/datasets/resource/15038).

Bibliographic records on each harvestmen taxon (except *Odiellus spinosus*) are listed in Merino-Sáinz and Anadón (2008, 2013) and Merino-Sáinz et al. (2013a). In the faunistic analysis, each species was considered in accordance with its general distribution as Iberian endemic, European element, or Holarctic element (see Merino-Sáinz and Anadón 2008). Specimens identified as *Trogulus nepaeformis* belong to a related undescribed species probably endemic to the Iberian Peninsula, according to Schönhofer and Martens (2010). However, distribution data on this undescribed species are not available; thus, the chorological type (European element) is retained to compare data with previous articles (Merino-Sáinz and Anadón 2008, 2013) and to test whether biases exist.

We conducted a hierarchical cluster analysis (group average clustering algorithm, see algorithm choice discussion in Clarke and Gorley 2006) on similarity matrices, in order to compare this subset with the data obtained through planned, standardised, and periodic samplings using pitfall traps in several locations in the north of the Iberian Peninsula (Merino-Sáinz and Anadón 2013). The inventory of each locality included all of the harvestmen species sampled using the pitfall traps in that locality; subset inventory included all of the harvestmen species present in the unplanned events collection. Only qualitative data on species presence, rather than abundance data, were used in the analysis, and the similarity matrices were calculated using a species-presence Sørensen index (Sørensen 1948) (only positive results, i.e., species present in a pair of inventories, incremental similarity between inventories, species absent from both inventories -double negative- don’t). Data were not standardised through sampling efforts, because the aims of the analysis were to compare the results of standardised sampling data with unplanned sampling data employing very different sampling and identification efforts. The analysis was carried out using the PRIMER V6 software (Clarke and Gorley 2006).

**Study extent description:** Harvestmen specimens included in the subset came from different localities in the Iberian Peninsula, at different distances from one another, and were collected at different dates between 1977 and 2011. Nonetheless, most of them came from the north of the Iberian Peninsula, and all of them came from the northern half (see Figure 1). The heterogeneity of the localities, most of which are only represented by a single sample or even only an isolated specimen, means that a general list of localities is not useful to short data exposition; rather, the locations are listed beside each specimen in Supplementary material 1 - Appendix A (supplementary file http://hdl.handle.net/10651/24734).

Harvestmen data obtained through planned collection using pitfall traps and deposited in the BOS Arthropod Collection included specimens collected from the Muniellos Biosphere Reserve between 2000 and 2002 (Merino-Sáinz and Anadón 2008) and from several locations in the provinces of Asturias (Muros, Oviedo, Villar), Cantabria (Vioño) and Pontevedra (Panjón) collected between 2009 and 2011 (Merino-Sáinz and Anadón 2013, Merino-Sáinz et al. 2013c). We also referred to published results of standardised pitfall samplings in western Asturias (Illano: Rosa García et al. 2009a, b, 2010a, b) for comparison purposes, as they were collected from the same areas (see Figure 1).

**Sampling description:** We studied a data subset of the harvestmen specimens in the BOS Arthropod Collection at the University of Oviedo that had been directly collected (by hand) on entomological field trips by students and lecturers from this department (listed in Supplementary material 1: http://hdl.handle.net/10651/24734, beside each specimen). This subset also included our own data obtained using diverse methods—collecting directly by hand, beating vegetation over an upturned umbrella, and using Berlese funnels, light traps, Malaise traps, and sieves; only 6% of collections used pitfall traps. Therefore, the specimens included in this study did not derive from harvestmen-targeted research projects, theses, or historical collections, but were collected at random with no prior sampling design.

## Quality control description

### Taxonomic identification

Specimens were identified by I. Merino-Sáinz using an Olympus SZX-ILLK200 stereoscopic microscope and the appropriate literature (Dresco 1948, 1954, Kraus 1961, Rambla 1959, 1967, 1973, 1976, 1980a, b, 1985, 1986, Sankey and Savory 1974, Martens 1978, 1982, Feliú 1981, Prieto 1990, 2004, Stol 2005, Pinto-Da-Rocha et al. 2007, Prieto and Fernández 2007, and Murienne and Giribet 2009).

### Digitisation quality control

The data quality control measures adopted throughout the digitisation process were described in the data-paper of the full dataset (Merino-Sáinz et al. 2013c) and in the digitisation workflow explication in Torralba-Burrial and Ocharan (2013). These controls included the validation and cleaning of geographic, taxonomic, and additional data associated with the harvestmen specimens (Merino-Sáinz et al. 2013c).

## Subset description

**Metadata language:** English

**Date of metadata creation:** 2014-02-05

**Hierarchy level:** Subset

**Metadata distribution:** http://www.gbif.es:8080/ipt/resource.do?r=bos-opi_unplanned_collection_events

**Format name metadata:** Ecological Metadata Language (EML) and HTML in web.

**Data distribution:** BOS-Opi dataset http://www.gbif.es:8080/ipt/archive.do?r=Bos-Opi

**Subset codes in dataset:** BOS-Opi codes 493-960.

**Publication date of data:** 2013-07-04

**Update police:** Subset will not be updated.

**Licences of use:** This BOS Arthropod Collection of University of Oviedo (Spain): Opiliones unplanned collection events subset, as part of BOS Arthropod Collection Dataset: Opiliones (BOS-Opi) dataset is made available under the Open Data Commons Attribution License: http://www.opendatacommons.org/licenses/by/1.0/.

## Data analysis

### Noteworthy records

**Figure 3.**
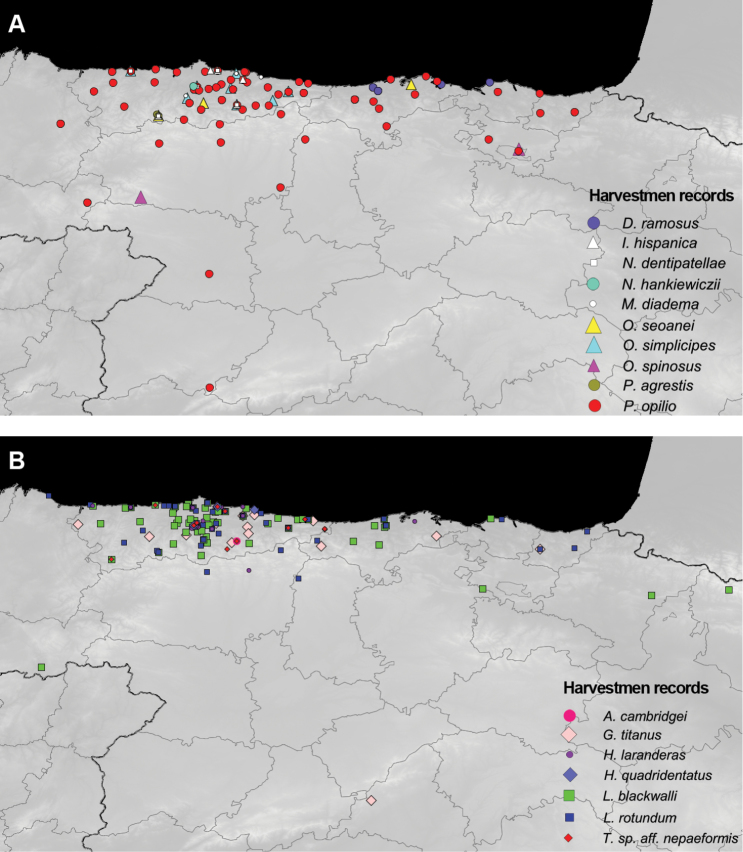
Distribution of harvestmen records in the unplanned collection events. **A** Ischyropsalididae, Nemastomatidae and Phalangiidae **B** Scleromatidae and Trogulidae.

In Figure 3, we have mapped the locations where each harvestmen species was found (listed in Supplementary material 1, http://hdl.handle.net/10651/24734), in order to facilitate rapid graphic assessment.

These records do not increase the harvestmen checklists for the provinces of Asturias and Cantabria (Merino-Sáinz and Anadón 2013), where planned, periodic, and standardised harvestmen-targeted sampling using pitfall traps have been carried out. However, the first records of five species are provided for other provinces that do not have this type of periodic targeted samplings. Table 2 lists the provinces with records for each species in this subset, indicating the first provincial records with an asterisk.

**Table 2.** Presence of each harvestmen species by province according to data included in this data subset.

	Orense	Lugo	Asturias	León	Zamora	Salamanca	Cantabria	Palencia	Burgos	Vizcaya	Guipúzcoa	Álava	Navarra	Huesca	Madrid
*Nemastomella dentipatellae*			X												
*Nemastoma hankiewiczii*			X												
*Trogulus nepaeformis*			X												
*Anelasmocephalus cambridgei*			X												
*Ischyropsalis hispanica*			X												
*Phalangium opilio*	X*	X*	X	X	X*	X*	X	X*	X	X*	X*	X*			
*Megabunus diadema*			X												
*Paroligolophus agrestis*			X												
*Odiellus simplicipes*			X												
*Odiellus seoanei*			X				X*								
*Odiellus spinosus*				X*					X*						
*Gyas titanus*			X				X				X				X
*Dicranopalpus ramosus*							X			X*					
*Leiobunum blackwalli*	X*		X				X			X		X	X	X	
*Leiobunum rotundum*		X	X	X			X	X*	X	X	X				
*Homalenotus laranderas*			X	X			X								
*Homalenotus quadridentatus*			X												
*Paramiopsalis* sp.			X												

* first records.

*Odiellus spinosus* is thus recorded for the first time in the provinces of León and Burgos. In Asturias, this species was found in Illano (Rosa García et al. 2009a, b, 2010a, b), but was absent in the pitfall samplings from Muniellos (Merino-Sáinz and Anadón 2008) and Central Asturias (Merino-Sáinz and Anadón 2013). Endemic *Odiellus seoanei* was confirmed in Cantabria with the detection of a male specimen. Previously, there had only been a single, old data record by Fernández-Galiano (1910) based on one immature specimen; that record was questionable due to the variability in taxonomic characters of immature *Odiellus* (Santos et al. 2008).

The first finding of *Leiobunum rotundum* in Palencia was not surprising, as this European species is widespread throughout the north of the Iberian Peninsula (Prieto and Fernández 2007); its absence in this province can be attributed instead to the shortage of data on Iberian harvestmen. Similar circumstances explain the first record of *Leiobunum blackwalli* in Orense; this species is widespread in the north of the Iberian Peninsula, with records in the neighbouring Portuguese districts of Vila Real and Bragança (Prieto and Fernández 2007).

The fact that this data subset includes the first records of *Phalangium opilio* in eight provinces is another example of the scarcity of data on harvestmen throughout the Iberian Peninsula. *Phalangium opilio* is a Holarctic species distributed throughout the peninsula from Galicia to Catalonia, with records in Portugal, Central Spain, and the Balearic Island (Kraus 1961), although specific information is lacking for several provinces. Therefore, it is safe to state that the digitisation of such unplanned collections has the potential to address existing gaps in knowledge.

The data subset also includes several records older than the first published records of some species in Asturias and Cantabria provinces, confirm earlier studies. Thus, we provide older records for three harvestmen species (*Megabunus diadema*, *Homalenotus laranderas*, and *Paroligolophus agrestis*) first reported in Asturias in 2008 (Merino-Sáinz and Anadón 2008) and for another two species reported in the same paper, though erroneously identified (*Odiellus simplicipes* specimens formerly identified as *Odiellus ruentalis*, and *Odiellus seoanei* specimens formerly identified as *Odiellus spinosus*; see Merino-Sáinz and Anadón 2013). Moreover, one *Homalenotus laranderas* female from Cantabria with data collected in 1982 was included (the first record in this province dates from 2009: Merino-Sáinz and Anadón 2013).

### Are there biases?

In the area covered by this data subset of Opiliones, systematic sampling has been conducted in seven localities; therefore, this subset should include the species caught in these samples (see Merino-Sáinz and Anadón 2013). The composition and frequency of species in this unplanned, non-harvestmen-targeted subset with no sampling design show differences from the data derived from periodic pitfall sampling in the north of the Iberian Peninsula (Rambla 1985, Rosa García et al. 2009a, b, 2010a, b, Merino-Sáinz and Anadón 2013). Since specimens were collected directly by hand, it was possible to obtain information about the habitat choice and habitat use of several harvestmen species, for which there was scarce data from pitfall traps.

**Figure 4.**
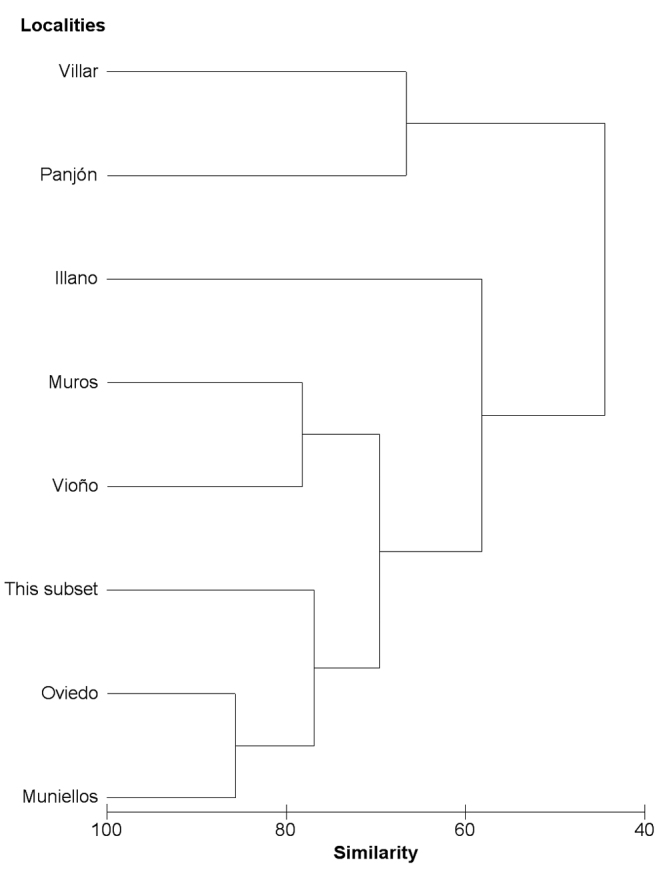
Cluster hierarchical analysis with harvestmen presence data from seven locations with planned collection events and this general subset.

Table 3 shows that this subset of unplanned collection events, with a similar number of specimens, includes only one species fewer than the systematic study on Opiliones from the Muniellos Biosphere Reserve (Merino-Sainz and Anadón 2008, 2009), the richest inventory of available studies on the area (Merino-Sáinz and Anadón 2013). Therefore, the unplanned collection events subset contains more species than any other listed study excluding Muniellos, even though the number of specimens is less than any of them by an order of magnitude. The next subset in the number of species, Oviedo, comprises 16 species with 15 times more studied specimens (Merino-Sáinz and Anadón 2013). Species richness and identity make this subset more similar to the inventories from Oviedo and Muniellos than to the remainder (see Figure 4). The differences with respect to the Muniellos Biosphere Reserve inventory are, on the one hand, the absence of three species – *Hadziana clavigera* (Simon), *Sabacon franzi* Roewer, and *Oligolophus hanseni* (Kraepelin) – from this subset and, on the other hand, the absence of *Homalenotus quadridentatus*, *Dicranopalpus ramosus*, and *Odiellus spinosus* from Muniellos (specimens identified as *Odiellus spinosus* in Muniellos are currently considered to belong to *Odiellus seoanei*: Merino-Sáinz and Anadón 2013). Differences with respect to the Oviedo inventory are due to four species that were not located in planned collection events using pitfall traps (*Paramiopsalis* sp., *Dicranopalpus ramosus*, *Odiellus spinosus*, and *Megabunus diadema*); the last one might be present in the area according to its distribution and habitat preferences (see Merino-Sáinz et al. 2013b). *Sabacon franzi* and *Hadziana clavigera* were likewise located in planned collection events in Oviedo. *Sabacon franzi* was also located with systematic standardised sampling in Muros and Illano; it coexists in the latter location with *Paroligolophus meadii* (O.P.-Cambridge), *Odiellus hansenii*, and *Iberosiro* sp. Bivort and Giribert, without data in this digitised subset. All species collected in the other locations using standardised sampling protocols were also included in this general subset.

**Table 3.** Number of harvestmen specimens and species with planned collection events (Oviedo, Muniellos, Illano, Muros and Vioño) and this subset.

	This subset	Oviedo	Muniellos	Illano	Muros	Vioño
Specimens	536	8452	770	1641	2687	2329
Species richness	18	16	19	14	13	12

Data sources of harvestmen data from planned collection events: Merino-Sáinz and Anadón (2008, 2013); Merino-Sáinz et al. 2013c, Rosa García et al. (2009a, b, 2010a, b).

These results also show that some taxa are not usually found in non-harvestmen-targeted (or soil entomofauna-targeted) samplings; this was the case for small, inconspicuous species that occupy edaphic niches throughout their entire life cycle (see previous comments on absent species). Instead, other species were better represented and appeared more frequently in the present subset, for example, large species with long legs and arboreal or shrub habits, at least during the adult phase. Taxa with these features comprised almost 56% of the species in this data subset, including the three most frequently captured species. Thus, the major abundance of adult specimens of *Phalangium opilio*, *Leiobunum rotundum* and *Leiobunum blackwalli* in the subset would be in line with observations in other geographic areas about vertical migration patterns throughout their life cycle (Todd 1949, Williams 1962, Allard and Yeargan 2005). The higher relative frequency of adults in these species is related to the use of active sampling methods, given that harvestmen spend more time in higher vegetation strata during their adult phase and are larger and more conspicuous than the immature specimens that predominate in soil and pitfall traps (e.g. Merino-Sáinz and Anadón 2013). However, *Homalenotus laranderas* and *Trogulus* sp. aff. *nepaeformis* are linked to edaphic habitats throughout their entire life cycle and present a cryptic coloration; each one species represents 4-5% of the specimens in this subset, occupying the fourth and fifth positions in the list of species in terms of the number of specimens collected (Table 1).

These biases are due to the differences in body size and life history of each harvestmen species and should be considered in biogeographic analyses with accidental occurrences (unplanned samples). In the particular case of this digitised subset, European elements comprised 50% of specimens, 39% were Iberian endemics, and 11% were Holarctic taxa—percentages which are slightly different from those resulting from pitfall trapping in the same area (Merino-Sáinz and Anadón 2013: 44%, 44%, and 12%, respectively). In both cases, namely the use of unplanned, non-standardised collections and the use of pitfall trapping, several Iberian endemic taxa with narrow niches (e.g., subterranean/hypogeous taxa) were absent; thus, these methods are not suitable for obtaining information about those taxa. A summary of advantages and problems arising from the digitisation of this subcollection of unplanned sampling events is provided in Table 4.

**Table 4.** Main observations on the advantages and problems arising from the digitisation of unplanned collections in the case study of Iberian harvestmen in the BOS Arthropod Collection.

Advantages	Problems
Less effort (identification, digitisation) needed: lower number of specimens than planned, periodical, pitfall samples	Some biases detected in harvestmen present in the subcollection (body size, life history, phases of life cycle)
Similar species richness	Does not provide full phenological data
Justification of the investment made to collect/house/study such collections	Not suitable for taxa with very narrow niches (e.g., subterranean/hypogean taxa)
Bridges knowledge gaps	

## Conclusions

A small subcollection of harvestmen from the north of the Iberian Peninsula, gathered using non-Opiliones-targeted sampling methods and in many cases by non-specialist collectors, presented a high species richness similar to planned, periodic, and costlier studies. This subcollection enabled us to extend our knowledge on the distribution of 18 species. The 536 specimens in the subset showed very interesting faunistic results, while less effort was exerted on identification and digitisation than in planned, periodic collection events using pitfall traps. The data subset contained six first provincial records of various species; *Phalangium opilio* locations in eight provinces without previous data were also provided. Nevertheless, we also detected some drawbacks to this type of data collection; collection was biased towards adults of larger species (with long legs or wide bodies) occupying shrubby or arboreal habitats, which may also affect the biogeographic analysis of the dataset. Nevertheless, this study highlights the importance of the general biodiversity collections in museums and at universities and the need to digitise their specimens, including the data from non-targeted, or unplanned, samplings, especially when poorly studied groups are involved. The digitisation of unplanned collections can help to justify the investments made to collect, house, and study such collections. Moreover, it is important to keep in mind that most of the collections at the university/museum, NGO, and amateur scientist levels are not comprised of data collected through planned events, but mainly through unplanned events. The digitisation of such unplanned collections has great potential to (1) bridge gaps in existing knowledge, and (2) strengthen existing understanding about the status of biodiversity.

## Supplementary material

Supplementary material 1 Harvestmen specimens included in this unplanned collection events subset. doi: 10.3897/zookeys.404.6520.app1 Data type: Specimens data. Explanation note: Alternative link for download: http://hdl.handle.net/10651/24734 This dataset is made available under the Open Database License (http://opendatacommons.org/licenses/odbl/1.0/). The Open Database License (ODbL) is a license agreement intended to allow users to freely share, modify, and use this Dataset while maintaining this same freedom for others, provided that the original source and author(s) are credited.Izaskun Merino-Sáinz, Antonio Torralba-Burrial, Araceli Anadón
