# Supplementary material for: The relevance, biases, and importance of digitising opportunistic non-standardised collections: A case study in Iberian harvestmen fauna with BOS Arthropod Collection datasets (Arachnida, Opiliones)
Source: Zookeys. 2014 Apr 24;(404):71–89. doi: 10.3897/zookeys.404.6520 (PMC4023260; doi:10.3897/zookeys.404.6520)
Supplement: Supplementary material 1 — Harvestmen specimens included in this unplanned collection events subset. [file zookeys-404-071-s001.pdf]

**Merino-Sáinz I, Torralba-Burrial A, Anadón A (2014) The relevance, biases, and importance of digitising opportunistic non-standardised collections: A case study in Iberian harvestmen fauna with BOS Arthropod Collection datasets (Arachnida, Opiliones).** ***ZooKeys*.**

**Appendix A.** Harvestmen specimens included in this unplanned collection events subset (metadata available at http://www.gbif.es:8080/ipt/resource.do?r=bos-opi_unplanned_collection_events). Specimens are deposited in the Opiliones subcollection in the University of Oviedo BOS Arthropod Collection (codes BOS-Opi 493-960). Species are listed according to taxonomic order following Pinto-Da-Rocha *et al.* (2007). All subcollection data are available in a DarwinCore Archive format in the data-paper Merino-Sáinz et al. 2013c, or through the GIBF data-portal (Universidad de Oviedo 2013-, http://data.gbif.org/datasets/resource/15038), including this subset. If you want a machine reading format or reuse the data is easier from that archive, extracting the registers with the BOS-Opi codes 493-960. MGRS coordinates (96% georeferencing rate) obtained by retrospective georeferencing (see Chapman and Wieczorek 2006) using digital cartography (GOOGLE EARTH, IBERPIX) are provided here (these are in decimal degrees in the DarwinCore Archive: Merino et al. 2013c). Abbreviations showing the sampling method: B = beating of vegetation, D = direct, by hand, P = pitfall trap, U = upturned umbrella, S = sieve.

**Suborden Eupnoi**
**Family Phalangiidae**
**Subfamily Dicranopalpinae**
**1.- *Dicranopalpus ramosus* (Simon)**

**Cantabria:** Laredo: Laredo, camping (30TVP6602706378): 02.08.09, D, 1♀ (I. Merino Sáinz leg.). Santillana del Mar: Santillana del Mar, wall of zoo park (30TVP1065103746): 08.03, D, 2♂♂, 1♀ (I. Merino Sáinz leg.); 24.09.04, D, 2♂♂. Torrelavega: Torrelavega, wall (30TVP1478300040): 17.08.11, D, 1♂, 1♀; 18.08.11, D, 2♀♀; 23.08.11, D, 2♂♂ (I. Merino Sáinz leg.).

**Vizcaya**: Górliz: Górliz, *Quercus ilex* forest (30TWP058087): 23.09.11, D, 1♂ (J. Alameda leg.).

Remarks: First record to Vizcaya.

**Subfamily Oligolophinae**
**2.- *Paroligolophus agrestis* (Meade)**

**Asturias:** La Felguera: field: 04.02.87, D, 1♂ (M. Cruz Súarez leg.). Quirós: Agüera (30TTN58): 30.10.86, D, 1♂, 2♀♀ (L. Riestra and Celsa F. San Narciso leg.); 30.10.89, D, 2♀♀ (Guillermo Barroso leg.). Somiedo: Pola de Somiedo (29TQH27): Anguino viewpoint (29TQH2204376117): hazel forest: 16.09.10, B, 1♀ (I. Merino Sáinz leg.); road border (29TQH2329374818): 16.09.10, D, 1♀ (I. Merino Sáinz leg.); field near river (29TQH2329374818): 17.09.10, D, 2♂♂, B, 1♀ (I. Merino Sáinz leg.); riparian forest (29TQH2329374818): 17.09.10, D, 1♂, 4♀♀ (I. Merino Sáinz leg.).

***Paroligolophus* sp.**

**Asturias:** Sobrescobio: Soto de Agues (30TTN9951586219): 10.08.12, D, 2JJ (Testing Biodiversidad 2012 leg.), 11.08.12, D, 1J (Testing Biodiversidad 2012 leg.), 12.08.12, S, 2JJ (Testing Biodiversidad 2012 leg.).

**3.- *Odiellus simplicipes* (Simon)**
**Asturias:** Cangas de Onís: Vega de Cien (30TUN2883990372): 01.06.94, D, 1♂ (Roberto Simal leg.). Colunga: hayedo La Biescona (30TUP21311): 29.04.86, D, 1♂ (José Luis Acuña leg.). Nava: Fuensanta (30TTP90): 15.08.90, D, 2♂♂ (A. Busto leg.). Onís: Avín (30TUN4169799847): 23.10.91, D, 1♂ (M. Menéndez Artime leg.). Oviedo: monte Naranco (30TTP6756607919): 05.93, D, 1♂ (M. Echevarría leg.); pine: 07.08.06, D, 1♂ (Laura González. Sánchez leg.). Quirós: Pedroveya (30TTN5914594882): 29.01.94, D, 1♂ (Ana Benito leg.). Sobrescobio: Soto de Agues (30TTN9951586219): 10.08.12, D, 1♂ (Testing Biodiversidad 2012 leg.). Valdés: Luarca (29TPJ991241): 23.10.90, D, 1♂ (C. Viña leg.).

**4.- *Odiellus seoanei* (Simon)**
**Asturias:** Mieres: Cenera (30TTN720891): meadow: 22.10.04, D, 2♂♂ (Jennifer García Álvarez and Paula Vallejo leg.). Somiedo: Pola de Somiedo: road border (29TQH2329374818): 16.09.10, D, 2♂♂ (I. Merino Sáinz leg.); Anguino viewpoint: hazel forest (29TQH2329374818): 16.09.10, U, 1♂ (I. Merino Sáinz leg.).
**Cantabria:** Marina de Cudeyo: Agüero_(30TVP4176406247): meadow: 04.08.05, D, 1♂ (Sergio Álvarez leg.).
Remarks: Presence confirmation from Cantabria.

**5.- *Odiellus spinosus* (Bosc)**
**Burgos:** Condado de Treviño (30TWN2961831759): Aguillo: 08.92, D, 1♂ (Virginia Zaldíbar leg.).
**León:** Truchas: Truchas (29TQG1153981823): 28.08.88, D, 1♂ (Carmen de Miguel leg.).
Remarks: First records from Burgos and León provinces.

***Odiellus* spp.**
**Asturias:** Aller: Aller, field: 06.04.08, D, 1♀ (M. Menéndez Fernández leg.). Cangas del Narcea: R.B. Muniellos (29TPH86): 12.06.88, D, 1♀ (G.E.P. leg.); 17.05.11, T, 9JJ (I. Merino Sáinz, A. Anadón and G. Giribet leg.). Corvera: Molleda (30TTP6449723595): 13.06.95, D, 1♀ (Sonia Cabo leg.). Cudillero: Cudillero (29TQJ3029526988): 13.11.04, D, 1♀ (Pilar Aranguren leg.). Gijón (30TTP82): garden: 03.07.86, D, 1♀ (Alex Merino leg.); wall: 25.10.92, D, 1♀ (A. Fernández García leg.); Somió (30TTP8835924338): 07.07.93, D, 1♀ (Guillermo G. Soto leg.); 24.08.93, D, 1♀ (Guillermo G. Soto leg.). Illano: research center El Carbayal SERIDA (29TPJ69600091): 16.05.11, D, 1J (I. Merino Sáinz, A. Anadón and G. Giribet leg.); La Montaña (29TPH7223398722): 16.05.11, S, 1J (I. Merino Sáinz, A. Anadón and G. Giribet leg.). Mieres: Mieres (30TTN7456392155): 25.08.98, D, 1♀ (Ana Pérez leg.); Cuna: 18.06.93, D, 1♀ (R. Sanz leg.); recreative area El Canto (30TTN720891): 26.10.01, D, 1♀ (A. Hevia leg.). Onís: Avín (30TUN4169799847): 21.10.91, D, 1♀ (Sara R. Fernández Fernández leg.); 22.10.91, D, 1♀ (A. Fernández Ojanguren); 23.10.91, D, 1♀ (Covadonga Isla leg.). Siero: Tiñana (30TTP7677405356): 19.04.94, D, 1♀ (Aurora F. leg.). Sobrescobio: Soto de Agues (30TTN9951586219): 11.08.12, D, 1J (Testing Biodiversidad 2012 leg.). Valdés: Luarca (29TPJ991241): 28.10.94, D, 1J (C. Váquez leg.); linden leaves: 26.10.94, D, 1♀ (P. Valdés García leg.); 11.05, D, 1♀ (M.A.G. Priede leg.).
**Asturias/León:** Puerto de Vegarada: 21.04.86, D, 1♀ (Andrés Fernández leg.).
**Burgos:** Castillo del Val (30TVM5181584676): 17.08.86, D, 2JJ (J.A. Soto and F.B. leg.).

**León:** León (30TTN8940219389): 24.05.90, D, 1J (M. Papazsurtundro leg.). Sagahún (30TUM3273893282): house: 04.07.87, D, 1J (J. Alperi Villar leg.).
**Palencia:** Fuente del Rey: on bush: 16.06.86, D, 2JJ (L. Carro and Mercedes Torres Michelena leg.).

**Subfamily Phalangiinae**
**6.- *Phalangium opilio* Linnaeus**
**Álava:** Kuartango: Zuazo (30TWN04): 07.95, D, 1♀ (R. Ocharan leg.). Sierra Badaya: 07.10.95, D, 1♀ (R. Ocharan leg.).
**Asturias:** Cabrales: Arangas de Cabrales (30TUN5407898589), garden: 08.83, D, 1♂, 1♀ (M. Antonia Díaz leg.). Cangas de Onís: Cangas de Onís (30TUP20): 01.07.89, D, 1J (Javier Gancedo leg.); Covadonga (30TUN3338297117): 09.93, D, 1♂ (Juan José Castro leg.). Cangas del Narcea (29TPH98): 28.10.96, D, 1♂ (M. García López leg.). Carreño: Carreño (30TTP729: 25.08.91, D, 1♂ (M. Jesús González leg.). Caso: Pendones, Tiatordos (30TUN18): 04.09.93, D, 1♀ (Pablo Prado Aller leg.); Caleao (30TUN0388380994): 28.05.08, D, 1♂ (J. Aladro leg.). Cudillero: Novellana (29TQJ1924327086): 22.07.91, D, 1♂, 1♀ (David Álvarez Fernández leg.). Gijón: Gijón (30TTP829): 15.09.95, D, 1♂ (Eduardo Díaz leg.); San Martín (30TTP829): 10.07.83, D, 1♀ (C. Martínez Pastor leg.). Gozón: Nembro: La Vega Susacasa (30TUP5078610928): 26.07.97, D, 1♀ (R. Rosa García leg.). Illano: research center El Carbayal (SERIDA) (29TPJ69600091): 16.05.11, D, 3JJ (I. Merino Sáinz, A. Anadón and G. Giribet). La Felguera: meadow: 22.08.87, D, 1♂ (Cruz Súarez leg.). Lena: Pola de Lena (30TTN6999882401), meadow: 01.05.88, D, 1♀ (M. A. Díaz Díaz leg.); 08.08.92, D, 1♂ (Julia Argüelles leg.). Llanes: Llanes (30TUP57920089199): 21.09.92, D, 1♀ (Conchita Gálvez leg.). Nava: Nava (30TTP9674203699): 28.07.83, D, 1♂, 1♀ (Pilar Gándara leg.). Navia: Navia (29TPJ8401123389): 08.86, D, 1♂ (Fidela Pérez leg.); 09.86, D, 1♂ (Luisa Súarez leg.). Onís: Avín (30TUN4169799847), heather: 23.10.91, D, 1♀ (Cristina Molina leg.), 24.10.91, D, 1♂ (Cristina Molina leg.). Oviedo: Oviedo (30TTP60): 08.09.86, D, 1♂ (B. Aguado leg.), 07.08.89, D, 1♂ (S. Pascual leg.), 08.03.91, D, 1♂ (Susana F. San Narciso leg.), 11.05.91, D, 1♀ (E. García leg.), 15.11.93, D, 1♂ (Begoña Galán leg.); Armeo: 04.09.09, D, 1♂ (A. Azumendi leg.); El Cristo (30TTP6721304389): 21.08.83, D, 1♂ (A. Barahona leg.); monte Naranco (30TTP6756607919): meadow: 18.06.86, D, 1J (Pilar Rodríguez. Pérez leg.); 06.04.87, D, 1♀ (Fidela Pérez leg.); Parque de Invierno (30TTP6920203656): 26.06.11, D, 1J (Omar Cots leg.). Ponga: Medandi (30TUN28): 07.08.04, D, 1♀ (D. Outomuro leg.). Quirós: Quirós (30TTN58): 30.10.86, D, 1♂ (Celsa Fernández San Narciso leg.). Riosa: cima el Angliru (30TTN606901): 21.09.10, D, 1♂, 1♀ (Ramón Barrial leg.). Salas: Las Gallinas (29TQJ1886912488), meadow: 08.06, D, 1♀ (Miguel Garredo leg.). San Martín del rey Aurelio: Santa Bárbara (30TTN8737892618): 03.09.92, D, 1♂, 1♀ (Antonio Fernández leg.). Siero: Careses (30TTP8713110158): ericas: 26.08.86, D, 1♂ (Alfredo González leg.); monte La Parte (30TTP8300206544): 06.08.94, D, 1♀ (J.C. Fernández Rodríguez. leg.); Tiñana (30TTP7677405356): 10.85, D, 1♀ (Isabel F. Súarez leg.). Sobrescobio: Soto de Agues (30TTN9951586219):10.08.12, D, 1♀ (Testing Biodiversidad 2012 leg.). Soto del Barco: Soto del Barco (29TQJ3644523994): 09.97, D, 1♂ (Rebeca G. leg.). Teverga: Puerto Ventana (29TQH4396571615): D, 2♂♂ (F.A. Fernández-Álvarez leg.). Valdés: Luarca (29TPJ991241): 25.10.89, D, 2JJ (J. Garai leg.), Pinewood: 23.10.90, D, 1♂ (G. Mutuberría leg.), 27.10.93, D, 1♀ (Roberto Simal leg.); Carlangas River: 02.11.95, D, 1J (M. Magdalena leg.). Villaviciosa: Villaviciosa (30TUP01): 14.06.91, D, 1♀ (Omar Cots leg.); Cordal de Peón (30TTP91), recreative area: 23.07.88, D, 2♂♂ (Carmen de Miguel leg.); Rodiles

(30TUP0769422814): meadow: 18.10.85, D, 1♂, 1♀ (Esther Orviz Díaz leg.), 18.10.85, D, 2♂♂ (Luis Orvíz Díaz leg.); Sietes (30TUP0836111498): 24.07.95. D, 1♀ (Gonzalo Alonso leg.); Tazones: La Atalaya (30TUP0646923495): D, 1J (F. Súarez Piñera leg.). Villayón: Carrio (29TPJ8515310942): meadow: 22.08.08, D, 1♀ (Graciela Súarez leg.).
**Burgos:** Condado de Treviño: Aguillo (30TWN2961831760): 08.92, D, 1♀ (V. Zaldívar leg.). Gumiel de Hizan: house: 29.09.82, D, 1♂ (Encarna Martín leg.). Edeja Montija: 20.09.90, D, 1♂, 1♀ (B. Baranda leg.).
**Cantabria:** Arnuero: Isla (30TVP5400215695): inside house: 12.08.86, D, 1♀ (Cristina Rodríguez Cabello leg.). Cieza: Collado de Cieza (30TVN1094888019): 26.07.96, D, 1♂ (C. Marcano leg.). Las Rozas de Valdearroyo: Bimón (30TVN2105560093): wall: 24.10.04, D, 1♂ (Enol García leg.). Molledo: Santa Cruz de Iguña (30TVN1532179823): 07.86, D, 1♀ (Carmen Gutiérrez leg.). Piélagos: Liencres (30TVP2506312450), playa: 04.08.82, D, 1♂, 1♀ (Luis Juli leg.). Riotuerto: puerto Alisas (30TVN49): 03.09.88, D, 1♂ (V. L. Doriga leg.). Ruente: Monte Ucieda (30TUN9579690531): 04.08.95, D, 2♂♂, 2♀♀ (Juan González. Rodríguez. leg.). Santoña: Santoña (30TVP6303310120): 29.07.93, D, 1♀ (Argilea Ceberio leg.).
Sin localidad: Cantabria, caseríos: 21.08.94, D, 1♀ (M.J.Villegas leg.).
**Guipúzcoa:** Bergara: Bergara (30TWN4692674727): 26.07.82, D, 1♀ (M. J. Arrayago leg.). Tolosa: Tolosa (30TWN75054759759: 17.07.93, D, 2JJ (Lukas Arbeloa leg.); 15.04.95, D, 1♀ (Alberto Luengo leg.). Valamana, tree: 03.07.88, D, 1♀ (Nerea Urizar leg.).
**León:** Oseja de Sajambre: Oseja de Sajambre (30TUN37): 16.07.87, D, 1♂ (F. Ballesteros leg.). Paredes: Murias (29TQH24): 27.07.97, D, 1♂ (Ovidio Rodríguez. leg.). Pola de Gordón: Pola de Gordón (30TTN84): 17.07.90, D, 1♀ (G. Rodríguez leg.). Sahagún: Sahagún (30TUM3273893282), camping: 27.08.91, D, 1♂ (Diego Fernández leg.). Valdeleón: on rocks: 30.06.87, D, 1♀; 30.07.87, D, 1♂, 1♀ (José Luis Vega Quizain leg.). Villamanín: Pendilla de Arbas (30TTN86): 08.11.92, D, 1♂ (M. Antoñanzas leg.).
**Lugo**: Baleira: Baleira (29TPH4307464127): 07.08.86, D, 2♀♀ (J. Rabal leg.).
**Orense:** A Veiga: Valdín, Villafernando (29TPG6744476545): 31.08.90, D, 1♂; 12.08.90, D, 1♀ (I. Ramón González. González. leg.).
**Palencia:** Velilla del Río Carrión: Valcobero El Cristo (Ceisto) (30TUN5432346426): 05.09.93, D, 1♀ (Ana C. Álvarez leg.).
**Salamanca:** Candelario: Candelario (30TTK6707472106): 02.08.92, D, 1♂ (Luis H. Torres leg.).
**Vizcaya**: Munguía: Laukariz (30TWN1207998291): 10.08.81, D, 1♂ (M. I. Saloña leg.). Ondarroa: Ondarroa (30TWN4659096550): 27.07.86, D, 1♀ (José Zulgica Bengoetxea leg.).
**Zamora:** Zamora (30TTL7079298668): 22.08.87, D, 1♂, 3♀♀ (I. M. Feital leg.).
Remarks: First records from Vizcaya, Guipúzcoa, Álava, Orense, Lugo, Zamora, Salamanca and Palencia.

**Subfamily Platybuninae**
**7.- *Megabunus diadema* (Fabricius)**
**Asturias:** Colunga: hayedo La Biescona (30TUP21311): 29.04.86, D, 2JJ (José L. Acuña and José I. Súarez Fdz leg.); La Isla (30TUP2003716642), near the beach: 01.05.86, D, 1♀ (Paloma Cuartas leg.). Santo Adriano: Tuñón (30TTN5810397679): on travertine: 29.04.11, D, 1♀ (F.A. Fernández-Álvarez leg.).
Remarks: Specimens belonging to material deposited in BOS Arthropod Collection commented in Merino-Sáinz *et al*. (2013a).

**Family Sclerosomatidae**
**Subfamily Gyinae**
**8.- *Gyas titanus* Simon**
**Asturias:** Colunga: Colunga (30TUP11): 29.04.86, D, 1J (J.L. Súarez Fernández leg.). Gijón: Peón (30TTP82): cueva: 05.89, D, 1J (F.J. Pérez Barbería leg.). Llanes: Purón River (30TUP6231907135): 03.06.03, D, 1J (I. Merino Sáinz leg.). Oviedo: Oviedo (30TTP609): 27.05.95, D, 1♀ (Puri Pérez leg.); Ules (30TTP66090691), meadow: D, 1♂ (M. Ojel leg.). Piloña: Infiesto: (30TUP0849902012), cave entrance: 16.06.82, D, 1♂ (Luis A. Calderón leg.); Riofabar (30TUN0913394404): fallen leaves: 16.04.03, D, 1J (J. Dugnol leg.). Quirós: Pedroveya, ruta las Xanas (30TTN5855594748): near river: 21.09.10, D, 1♂, 1J (F. A. Fernández-Álvarez leg.). Siero: mount La Parte (30TTP8300206544): 16.04.10, D, 1♂ (J.M. Súarez leg.). Sobrescobio: Sobrescobio (30TTN98), 16.04.83, D, 1J (Ernestina Cobo leg.). Taramundi: Bres (29TPJ5680103681), meadow: 16.04.87, D, 1J (S.M. Santaeufemia leg.). Piloña: Espinedo, monte Moriacos (29TQH1508692053), fallen leaves: 06.11.93, D, 1♀ (Jorge Llamazares leg.). Villaviciosa: Peón (30TUP01): 01.05.89, D, 2JJ.
**Cantabria:** Potes: Potes (30TUN6801579163): 07.86, D**,** 1♂ (F. González Álvarez leg.). Ramales de la Victoria: Vegacorredor (30TVN6229389438): 23.08.92, D, 1♂ (J. Zalama Tena leg.).
**Guipúzcoa:** Bergara: Bergara (30TWN4692674727): 18.06.90, D, 1J (Nerea Aperribay Azpeitia leg.).
**Madrid:** El Escorial (30TVK09): garden: 15.09.85, D, 1J (J. González leg.).

**Subfamily Leiobuninae**
**9.- *Leiobunum blackwalli* Meade**
**Álava**: Latarón: Fontecha: 19.03.91, D, 1♀ (Sandra Ortíz leg.).
**Asturias:** Ablanedo**:** 26.10.94, D, 1♀ (Puri Pérez leg.). Avilés: Avilés **(**30TTP62): 09.92, D, 2♀♀ (O. Barquero leg.); 20.08.93, D, 1♀ (Yolanda Bango M. leg.); 26.08.93, D, 1♂, 1♀ (Paqui Morán leg.); Valparaíso (30TTP62): 24.09.92, D, 1♀ (Laura Rodríguez. González. leg.). Candamo: Sandiche (29TQJ3799612073): 22.03.89, D, 1♀ (J. I. Arias Álvarez leg.). Cangas de Onís: Cangas de Onís (30TUP20): 01.07.89, D, 1♀ (Javier Gancedo leg. Cangas del Narcea: R.B. Muniellos (29TPH86): ash gallery forest: 10.05.01, D, 2JJ (Ocharan *et al.*, 2003 leg.). Carreño: Guimarrón (30TTP72): 20.08.90, D, 1♀ (M. Jesús González. leg.); Candás (30TTP7670530130): 15.05.93, D, 1♀ (Sonia González. leg.).Caso: Campo (30TUN0939683790), muro: 12.08.08, D, 1♀ (Lidia Aladro Calvo leg.). Castrillón: Castrillón (29TPJ70): 06.90, D, 2♂♂ (C. Álvarez leg.); Salinas (30TTP6092129230): 19.08.82, D, 1♂ (José Ramón García leg.); Valboniel (30TTP5937226633): 06.90, D, 1♀ (C. Álvarez leg.). Cudillero: Lairín (Novellana) (29TQJ1866427674): 110.07.93, D, 1♀ (Ricardo P. Fernández leg.). Gijón: Gijón (30TTP82): 22.05.89, D, 1♀ (Fdo Álvarez leg.), 09.08.95, D, 1♀ (Eduardo Díaz leg.); Ceares (30TTP8528722189), meadow: 30.10.86, D, 1♂ (Jesús G. Durán leg.); Deva (30TTP9081820194): 03.08.95, D, 1♀ (C. Vázquez leg.); Granda (30TTP8510420181), vegetable garden: 01.11.07, D, 1♀,11.07.09, D, 1♂ (V. I. Álvarez leg.); Mareo (30TTP8274619511): 17.05.93, D, 1♂, 1♀ (Ángeles Martínez leg.); San Martín (30TTP82): 10.07.83, D, 1♀ (L. Martínez Pastor leg.); Xivares (30TTP82): 30.08.94, D, 1♂ (M. Cossío Aranceta leg.). Grado: Grado (29TQJ3696807826): 21.03.89, D, 1♂ (J. I. Arias Álvarez leg.); El Rodaco (29TQJ30), jardín: 12.07, D, 1♂ (A. Quevedo Rojo leg.). Illano: Illano, wall: 08.12.02, D, 1♀ (C. López leg.). Las Regueras: Biedes (30TTP6249812285): 22.11.10, D, 1♀ (Javier Díaz leg.); El Escamplero

(30TTP6096007789), bushes: 24.03.07, D, 1♀ (Pedro Arias González. leg.). Lena: Espinedo (30TTN6961971604), meadow: 03.08.07, D, 1♂ (Jesús Ortíz leg.). Llanera: Soto de Llanera (30TTP7550113433): 25.10.95, D, 1♀ (Sergio Álvarez leg.). Llanes: Celorio (30TUP5344709883): 07.08.94, D, 1♂ (Puri Pérez leg.); Llanes (30TUP5792008919): 20.10.91, D, 1♂ (Antideo Rodríguez. leg.), 03.09.93, D, 1♀ (Conchita Gálvez leg.); Posada (30TUP4925409561): 07.88, D, 1♂ (Paulino Bárcena leg.). Langreo: La Felguera (30TTN89), meadow: 25.05.87, D, 1♂ (M. Cruz Súarez leg.). Mieres: Bustiello (30TTN7494185562): vegetable garden: 06.82, D, 1♀ (José Fernández leg.); Cenera (30TTN720891): 14.11.04, D, 1J (I. Hidalgo leg.), 10.10.08, D, 1♂ (Irene Solís leg.); Les Vallines (30TTN7195585342): 20.08.81, D, 1J (E. Sierra Gurez leg.); Turón (30TTN5810397679): 28.01.02, D, 1♀ (María López leg.); Vega la Fonte (30TTN7456392155): 05.05.82, D, 1♀ (A. Abril leg.). Muros de Nalón: Muros de Nalón (29TQJ3378725197):15.06.89, D, 1♀ (Ángel Valdés leg.). Noreña: Noreña (30TTP8077008148): 15.08.89, D, 1♂ (S. Pascual leg.). Olloniego: La Manzaneda (30TTN7087499196): D, 1♂ (Pablo Díaz leg.). Onís: Avín (30TUN4169799847): 22.10.91, D, 1♀ (Juan Zalama leg.); 23.10.91, D, 2♀♀ (M. Menéndez Artime and Julia Rodríguez. leg.). Oviedo: Ajuyán (30TTP6541010483): 16.10.09, P, 1J (I. Merino Sáinz and A. Anadón leg.), 30.10.09, P, 1J (I. Merino Sáinz and A. Anadón leg.); Brañes (30TTP6421210582), stones: 19.11.04, D, 1♀ (Julia Díaz leg.); Oviedo (30TTP60): 28.10.85, D, 1♀ (Isabel F. Súarez leg.), 01.11.88, D, 2♀♀ (J. Villasante leg.), 14.03.89, D, 1♀ (Jorge González. leg.), 06.05.90, D, 1♀ (Jon Bereciartúa leg.), 21.06.93, D, 1♂ (A. Remacha leg.), 28.01.94, D, 1♀ (E. Rosal Fraga leg.), 14.03.96, D, 1♀ (Belén Sáinz leg.); Ules (30TTP66090691): 17.09.09, P, 1J (I. Merino Sáinz and A. Anadón leg.). Peñalba: grasses: 29.11.83, D, 1♀ (I. Bascarán leg.). Pravia (29TQJ3353719097), wall: 08.04.91, D, 1♂ (Antonio Martínez leg.); San Esteban (29TQJ3528126336): 24.08.93, D, 1♀ (C. Pavón Iturmendi leg.). Quirós (30TTN58), church: 15.10.86, D, 1♀ (M. Arbesú leg La Desgarrada pass (bear way¿senda del oso?) (30TTN58): D, 1♂, 1♀ (F.A. Fernández-Álvarez leg.). Ribadesella: Llovio (30TUP3364611688): 09.08.86, D, 1♂ (R. M. García leg.). Ribera de arriba: Tellego (30TTN6739198006): 15.10.85, D, 1♀ (Asun Prada leg.). Rioseco: 17.11.85, D, 1♀ (E. Pérez Antolín leg.). Siero: Pola de Siero (30TTP8447107708): 20.06.90, D, 1♂, 23.06.90, D, 1J (Ana Palacios leg.); Tiñana (30TTP7677405356): 15.11.93, D, 1♂ (Ana Benito leg.). Somiedo: Pola de Somiedo: Anguino viewpoint(29TQH2329374818): 16.09.10, U, 1♀ (I. Merino Sáinz leg.). Tapia de Casariego: Salave (29TPJ6831725133): house: D, 1♀, 1J (Damiana González. leg.). Teverga: La Torre (29TQH38), vivienda: 01.11.03, D, 1♂ (Zaida Fernández López leg.). Tineo: Fastias (29TQJ0698808845): 04.11.01, D, 1♂ (Almudena Súarez leg.). Valdés: Aquelclaro (29TPJ92): meadow: 11.08.95, D, 1♀ (Joaquín Pérez leg.); Luarca (29TPJ991241), meadow: 26.10.88, D, 2♀♀ (Claudia Martínez and E. C. Jiménez leg.), 20.10.89, D, 1♀ (S. Aguirre leg.), 24.10.89, D, 1♀ (Celestino González. leg.), path edtge: 23.10.91, D, 1♀ (M. A. Paredes leg.), 26.10.93, D, 1♂ (Argilea Cebeiro leg.), 30.10.95, D, 3♂♂, 1♀ (L. Frechilla and Belén Sáinz leg.), 02.11.95, D, 1♀ (M. Magdalena leg.); San Timoteo (29TPJ991241), meadow: 22.10.90, D, 1♀ (P. Velasco Vallejo leg.), 23.10.90, D, 1♀ (Ana Murguizo leg.); Esva River (29TPJ991241): 22.10.90, D, 1♀ (M. Teresa Bravo leg.); Negro River (29TPJ991241): 25.10.89, D, 3♂♂, 3♀♀, 1J (A. Anadón, Idoia Adanaga, I. Garai and I. Esteban leg.); Villar (29TPJ991241): 25.10.89, D, 1♂, 1♀, 1J (Clara Fernández Ochoa leg.), 22.10.90, D, 2♀♀, 1J (Hugo Mortera, A. M. González. Lozano and Diana Cifuentes leg.), 23.10.90, D, 2♂♂ (C. Villa and M. Isabel Síndrez leg.), 26.10.94, D, 1♀ (Brezo Martínez leg.), 30.10.95, D, 1♂ (Presen Ordiérez leg.); Otur (29TPJ9453123461): 17.03.07, D, 1♂

(Vanessa Oroza leg.). Villaviciosa: Rodiles (30TUP0769422814), meadow: 18.10.85, D, 1♀ (Luis Orníz Díaz leg.); Villaviciosa (30TUP01): 02.06.90, D, 1♂ (I. Riera leg.).
**Cantabria:** Cabuérniga: Cabuérniga (30TUN9412584392): 14.04.88, D, 1♀ (Olivia Fraolillas leg.). Miengo: Miengo (30TVP1957408692), ¿barn?stable: 07.07.83, D, 1♂ (M. Ramos leg.). Molledo: Santa Cruz de Iguña (30TVN1532179823): 07.87, D, 1♂ (Ana M. González. Antuña leg.). Piélagos: Vioño (30TVP2148901539): 04.08.03, D, 1♀, 08.04.04, D, 2JJ, wall: 18.03.08, D, 1♀, 05.04.08, D, 1♀ (I. Merino Sáinz leg.); Zurita (30TVN1993499931): 19.01.94, D, 1♀ (Laura Alonso leg.). Reocín: Puente San Miguel (30TVP1183101371): 14.05.94, D, 1♀ (Ángeles Gómez leg.).
**Huesca:** Canfranc: Canfranc (30TYN0261332262): 17.08.07, D, 1♂ (Pilar J. B. leg.).
**Navarra:** Lumbier: Lumbier (30TXN3903724646): 25.09.08, D, 1♀ (Idoia Villate leg.).
**Orense:** Verín: Verín (29TPG2957344345): 23.07.82, D, 1♀ (Generosa Álvarez leg.).
**Vizcaya:** Górliz: monte Ermua (30TWP0633208583): 23.09.11, D, 1J (I. Merino Sáinz leg.).
REMARKS: First record from Orense.

**10.- *Leiobunum rotundum* (Latreille)**
**Asturias:** Amieva: Amieva: 02.08.96, D, 1♂ (Mónica Gutiérrez leg.). Avilés: Avilés (30TTP62): 24.07.83, D, 1♀ (C. Súarez leg.); 03.08.90, D, 1♂ (C. M. Álvarez leg.); 20.08.93, D, 1♀ (Yolanda Bango M. leg.); Valparaíso (30TTP62): 24.09.92, D, 1♂ (Laura Rodríguez González. leg.). Cangas de Onís: Cuviella (30TUP2400006264): 02.09.92, D, 1♂ (Luis Cabo Pérez leg.). Cangas del Narcea: Cangas del Narcea (29TPH98), tree: 04.11.06, D, 1♀ (Daniel Rodríguez. Fernández leg.). Carreño: Candás (30TTP7670530130): 07.05.93, D, 1♂ (Sonia González. leg.). Corvera: Colunga: La Isla (30TUP2003716642): D, 1♂ (Alfredo Díaz leg.); Lastres (30TUP1645820371): stable: 01.06.90, D, 1♂ (Ramón Garrido leg.). Corvera: Cancienes (30TTP6726021291): wall: 22.10.05, D, 1♀ (Laura Perceño Fernández leg.). Cudillero: Cudillero (29TQJ3029526988): 18.11.01, D, 1♂ (Carmen Llera leg.); Lamuño (29TQJ2596726980): inside house: 08.85, D, 1♂ (J. M. Rico Ordás leg.). Gijón: Deva (30TTP82): 04.09.83, D, 1♀ (Higinio Bernardo leg.); Gijón (30TTP82): 05.82, D, 1J (Susana Junquera leg.); Porceyo (30TTP8080119952): 02.11.95, D, 1♀ (Fernando Fernández Álvarez leg.). La Felguera, meadow: 25.05.87, D, 1♀ (M. Cruz Súarez leg.). Langreo: Reguerines (30TTN89): 29.07.92, D, 1♀ (Pilar Iglesias leg.); Riaño (30TTP7962800799): 20.07.93, D, 1♂ (A. Noval leg.). Laviana: Felguerina: 31.08.07, D, 1♂ (Pamela García leg.). Lena: Pola de Lena (30TTN6999882401): 05.08.92, D, 1♀ (Julia Argüelles leg.). Llanes: Llanes (30TUP5792008919): 02.11.02, D, 1♂ (Ana Fernández Ordíz leg.). Mieres: Cenera (30TTN720891): 10.10.08, D, 1♂ (Álvaro Penaos leg.); 22.10.04, D, 2♂♂ (Pedro Rodríguez. Pérez and Susana Fernández leg.); La Vegona (30TTP8300206544): 06.12.07, D, 1♀ (Lucía Cabeza leg.). Onís: Avín (30TUN4169799847): 21.10.91, D, 2♂♂ (Sara R. Fernández Fernández and Jesús M. Burgués leg.); 22.10.91, D, 2♂♂ (M. José Goicoechea and A. Fernández Ojanguren leg.); 23.10.91, D, 1♂, 1♀ (I. Oliveros and J. S. Moro leg.). Oviedo: Latores (30TTP6642603004): 10.85, D, 1♂ (Gloria Garzón leg.); mount Naranco (30TTP6756607919), tronco tree: 22.05.88, D, 1♂ (Nerea Urizar leg.), 03.11.88, D, 1♂ (T. Montoto leg.), 27.08.08, D, 1♂ (Lucía Cabeza leg.), 20.09.10, D, 1♂ (Luis García leg.); Oviedo (30TTP60): 24.03.86, D, 1♀ (M. Gregoria García leg.), 03.09.88, D, 1♂,1♀ (M. José Ruíz leg.), 20.03.90, D, 1♀ (I. Riera leg.); San Claudio (30TTP6337403899): 08.08.86, D, 1♀ (Teresa Súarez González. leg.); Santa Ana de Abuli (30TTP7235605141): 15.10.85, D, 1♂ (Isabel F. Súarez leg.). Pravia: San

Esteban de Pravia (29TQJ3528126336): 24.08.93, D, 1♂ (C. Pavón leg.). Quirós: Quirós (30TTN58): 08.86, D, 1♀ (J. Villa Cienfuegos leg.), church: 15.10.86, D, 2♂♂, 1♀ (M. Nélida García Martínez and M. Arbesú leg.), 30.10.86, D, 7♂♂, 1♀ (Ana Iquierdo, Carmen R. Oleaga and Celsa F. San Narciso leg.), meadow: 05.05.87, D, 1♂ (Flor Rodríguez. leg.). Sama (30TTN89): 19.08.88, D, 1♀ (R. Edreandia leg.). Siero: mount la Parte (30TTP8300206544): 06.08.94, D, 1♀ (J. C. Fernández Rodríguez. leg.). Somiedo: Pola de Somiedo (29TQH2329374818): 04.11.97, D, 1♂ (Vanessa A. Faes Leg), gallery forest: 17.09.10, D, 1♂, 1♀ (J. Miñano and J. Benhadi leg.); Anguino viewpoint (29TQH2204376117), hazel forest: 16.09.10, U, 1♀, B, 1♂ (I. Merino Sáinz leg.). Sobrescobio: El Castrín, meadow: 21.11.02, D, 1♂ (Elisa García Alegre leg.); Soto de Agues, ruta del Alba (30TTN9951586219): 10.08.12, D, 4♂♂ (Testing Biodiversidad 2012 leg.). Tapia de Casariego: Tapia de Casariego (29TPJ6599826163): 20.08.93, D, 1♀ (Judith F. Casero leg.). Tineo: El Rodical (29TQH0958498632): 05.93, D, 1♂ (Elvira García leg.). Valdés: Luarca (29TPJ991241): L. Olaizola leg.), 23.10.89, D, 1♂ (Rosa Menéndez leg.), 14.10.90, D, 1J (Celestino González. leg.), 26.10.93, D, 2♂♂ (Pablo Prado Aller and Argilea Ceberio leg.); Villar (29TPJ991241): 01.11.89, D, 1♂ (Ángeles Lauriño leg.), 22.10.90, D, 1♂ (I. Urbaneta Blanco leg.), 31.10.95, D, 1♀ (Presen Ordiérez leg.), 01.11.95, D, 1♂ (M. Teresa Mieres Valdés leg.). Villaviciosa: Villaviciosa (30TUP01): 05.11.88, D, 1♂ (J. Villasante leg.).
**Burgos:** Gumiel de Hizán: 19.08.82, D, 1♀ (Encarna Martínez leg.).
**Cantabria:** Cillorigo de Liébana: Bedoya (30TUN68): 28.08.93, D, 1♂ (Ángela F. Roiz leg.). Miengo: Miengo (30TVP1957408692), barn?stable: 09.07.83, D, 1♀ (M. Ramos leg.). Piélagos: Vioño (30TVP2148901539): 04.08.03, D, 1♀ (I. Merino Sáinz leg.).
**Guipúzcoa:** Bergara: Bergara (30TWN4692674727): 23.07.82, D, 2♀♀ (M. J. Arrayago and José R. García leg.). Oúar: 06.07.88, 1♂ (Eva Aguirre leg.). Donostia: San Sebastián (30TWN89): 08.88, D, 1♀ (A. Ugeld leg.). Tolosa: Tolosa (30TWN7505475975): 15.04.95, D, 1♂ (Alberto Luengo leg.).
**León:** La Pola de Gordón: Geras (30TTN7403252815): 09.86, D, 1♀ (G. Barroso leg.). Oseja de Sajambre: Oseja de Sajambre (30TUN37): 16.07.87, D, 1♀ (F. Ballesteros leg.).
**Lugo:** Burela: Burela (29TPJ3215635556): 04.87, D, 1♂ (L. Palacios leg.).
**Palencia:** Velilla del Río Carrión: Velilla del Río Carrión (30TUN4898543197): 18.08.93, D, 1♀ (Ana C. Álvarez leg.).
**Vizcaya:** Bakio: Bakio (30TWP1554207956): 10.04.89, D, 1J (Ana Abio leg.).
Remarks: First record from Palencia.

***Leiobunum* sp.**
**Asturias:** Cangas del Narcea: San Pedro de Corias (29TQH0083986682): 18.05.12, S, 1J (I. Merino Sáinz, A. Anadón and G. Giribet leg.); R.B. Muniellos (29TPH86): 17.05.11, D, 1J (I. Merino Sáinz, A. Anadón and G. Giribet leg.). Oviedo: El Violeo de Brañes (30TTP6408): 27.03.09, P, 1J (I. Merino Sáinzy A. Anadón leg.). Siero: Pola de Siero (30TTP8447107708): 23.06.90, D, 1J (Ana Palacios leg.). Teverga: Ventana pass(29TQH4396571615): D, 1J (F.A. Fernández-Álvarez leg.).

**Subfamily Sclerosomatinae**
**11.- *Homalenotus laranderas* Grasshoff**
**Asturias:** Avilés: San Cristóbal (30TTP62): pine: 09.92, D, 1♀ (Susana Barquero leg.).Gijón: Gijón (30TTP82): 05.82, D, 1♀ (Susana Junquera leg.), 29.07.83, D, 1♀ (Nieves Alonso leg.), 10.08.83, 1♂ (P. Martínez leg.); San Martín (30TTP82): 10.08.83,

D, 1♀ (P. Martínez Pastor leg.); Somió (30TTP8835924338): 07.08.93, D, 2♀♀ (Guillermo G. Soto leg.). Langreo: Riaño (30TTP7962800799): 30.05.94, D, 1J (A. Noval leg.). Oviedo: Campiello (30TTP6861103201): 03.03.86, 1♂, 1♀ (J.A. Pis and Juan E. Ortíz leg.); El Cristo (30TTP6721304389): 03.08.88, D, 3♀♀, 1J (A.M. Navarro Incío leg.); Las Caldas (30TTP6304701605): 17.07.88, D, 1♀ (Paulino Bricena leg.); Oviedo (30TTP60): 26.05.00, D, 1J; Parque de Invierno (30TTP6920203656): 10.85, D, 1♂ (Gloria Garzón leg.). Peñamellera Baja: Panes, La Brañona (30TUN7124698104): 23.02.91, D, 1♂ (Loren P. Melero leg.). Siero: Lugones (30TTP7253609711): grassy path: D, 1J (A. Niembro leg.). Tapia de Casariego: Salave (29TPJ6831725133): 10.05.90, D, 1♂ (Damiana González leg.). Valdés: Luarca (29TPJ991241): 23.10.89, D, 3♀♀ (C. Álvarez, Diego Alvarado and Alberto Diego leg.), 10.95, D, 1♀ (J.L. Ortíz Suárez leg.). Villaviciosa: Villaviciosa (30TUP02): 07.05, D, 1♀ (Marcos Miñarro leg.); Porreo (30TUP02): 20.11.77, D, 1♂.
**Cantabria:** Medio Cudeyo: Solares (30TVP40): 05.08.82, D, 1♀ (Pilar Gándara leg.).
**León:** Boñar: Oville (30TUN0833353314): 20.07.83, D, 1♀ (A. Barahona leg.).

**12.- *Homalenotus quadridentatus* (Cuvier)**
**Asturias:** Colunga: Luces (30TUP1469621033), under stone: 26.06.90, D, 1♀ (Ramón Garrido leg.). Gijón: Peón (30TTP82), on moss: 20.12.86, D, 1♀ (R. Blanco leg.). Oviedo: Oviedo (30TTP60): 05.12.90, D, 1♀(E. García leg.).

***Homalenotus* sp.**
**Asturias:** Somiedo: Pola de Somiedo (29TQH2329374818): 18.05.11, S, 3JJ (I. Merino Sáinz, A. Anadón and G. Giribet leg.).

**Suborden Dyspnoi**
**Superfamilia: Ischyropsalidoidea**
**Family: Ischyropsalididae**
**13.- *Ischyropsalis hispanica* Roewer**
**Asturias:** Gijón: Peón (30TTP82), on moss: 20.12.86, D, 1♂ (Guillermo Barroso leg.). Cangas del Narcea: R.B.Muniellos (29TPH86), fresneda: 26.04.02, P, 1♀ (Ocharan *et al.*, 2003 leg.). Carreño: Pervera, barrio el Monte (30TTP7915825144): 11.94, D, 1♀ (David Martínez leg.). Somiedo: Pola de Somiedo (29TQH2329374818), road border: 16.09.10, D, 1♂ (J. Benhadi leg.), bosque ribereño bajo piedra: 17.09.10, D, 1♂, 1♀ (J. Benhadi leg.). Sobrescobio: Soto de Agues, ruta de Alba (cave) (30TTN9951586219): 12.08.12, D, 1♂, 1♀ (Testing Biodiversidad 2012 leg.). Villaviciosa: Peón (30TUP01), meadow: 29.10.88, D, 1♀ (A. C. Díaz leg.). Without locality: cave: 25.07.92, D, 1♂ (Félix González leg.).

**Superfamilia Troguloidea**
**Family Nemastomatidae**
**Subfamily Nemastomatinae**
**14.- *Nemastomella dentipatellae* (Dresco)**
**Asturias:** Gijón: Gijón (30TTP82): 22.05.89, D, 1♀ (Fdo Álvarez leg.). Oviedo: mount Naranco (30TTP6756607919): 12.02.10, P, 1♀ (I. Merino Sáinz and A. Anadón leg.). Somiedo: Pola de Somiedo (29TQH2329374818): 18.05.11, S, 1♀ (I. Merino Sáinz, A. Anadón and G. Giribet leg.). Sobrescobio: Soto de Agues (30TTN9951586219): 10.08.12, D, 1♀ (Testing Biodiversidad 2012 leg.), 11.08.12, S, 2♂♂, 1J (Testing Biodiversidad 2012 leg.). Valdés: Luarca, Negro River (29TPJ991241): 25.10.89, D, 1♀ (A. Anadón).

**15.- *Nemastoma hankiewiczii* (Kulczynski)**
**Asturias:** Oviedo: El Violeo de Brañes **(**30TTP6408): 27.0.09, P, 1♂ **(**I. Merino Sáinz and A. Anadón leg.)

**Family Trogulidae**
**16.- *Trogulus* sp. aff. *nepaeformis* (Scopoli)**
**Asturias:** Aller: Collanzo (30TTN9143777720): 18.05.89, D, 1♀ (Rolando Rodríguez Muñoz leg.). Cangas del Narcea: Muniellos (29TPH86): 20.11.92, D, 1♀ (Pilar Uría Tejera leg.). Cudillero: Novellana (29TQJ1924327086): 22.07.91, D, 1♀ (David Álvarez Fernández leg.). Gijón: Deva (30TTP9081820194); Gijón (30TTP82), meadow: 04.04.94, D, 1♀ (J.J. Porta Allende leg.), 20.05.09, D, 1♀ (Luis de Pedro leg.). Llanes: Poo (30TUP5569009432): 11.05.88, 2♀♀ (V. Alperi Villar leg.). Peñamellera Baja: Panes (30TUN7161997800): 09.11.90, D, 1J (Loren P. Melero leg.), La Brañona (Panes) (30TUN7124698104): 23.02.91, D, 1♂, 1J (Loren P. Melero leg.). Onís: Avín (30TUN4169799847): 23.10.91, D, 1♀ (J. M. Burgués leg.). Oviedo: Oviedo (30TTP60): 15.06.89, D, 1♀ (E. Eguilegor leg.), 16.06.89, D, 1♀ (I. Irizar leg.), mount Naranco (30TTP6756607919): 07.09, P, 5♂♂, 3♀♀ (I. Merino Sáinz and A. Anadón leg.). Sobrescobio: Soto de Agues (30TTN9951586219): 11.08.12, S, 1J (Testing Biodiversidad 2012 leg.).

**17.- *Anelasmocephalus cambridgei* (Westwood)**
**Asturias:** Sobrescobio: Soto de Agues (30TTN9951586219): 11.08.12, D, 1♂ (Testing Biodiversidad 2012 leg.).

**Suborden Cyphophthalmi**
**Family Sironidae**
**18.- *Paramiopsalis* sp. Juberthie**
**Asturias:** Illano: La Montaña (29TPH7223398722): 16.05.11, S, 7♂♂, 4♀♀, 1J (I. Merino Sáinz, A. Anadón and G. Giribet leg.).
